# Recurrent Pituitary Adenoma Causing Cushing’s Disease in a Patient With Lynch Syndrome

**DOI:** 10.7759/cureus.102414

**Published:** 2026-01-27

**Authors:** Vojtech Bares, David Netuka

**Affiliations:** 1 Medicine, First Faculty of Medicine, Charles University, Prague, CZE; 2 Neurosurgery and Neuro-oncology, Military University Hospital, Charles University, Prague, CZE

**Keywords:** cushing’s disease, cushing syndrome, endometrioid adenocarcinoma, lynch syndrome, msh6, pituitary adenoma, transsphenoidal surgery

## Abstract

Cushing’s disease (CD) is a severe endocrine disorder caused by an adrenocorticotropic hormone (ACTH)-secreting pituitary microadenoma. The diagnosis of CD remains one of the most challenging in endocrinology due to a wide array of symptoms caused by the extensive distribution of glucocorticoid receptors. Transsphenoidal surgery (TSS) is the first-line treatment, but recurrence following TSS is not uncommon. Repeat TSS and pituitary radiotherapy are among the second-line treatments of CD. Separately, Lynch syndrome (LS) is an autosomal dominant disorder caused by mutations in DNA mismatch repair (MMR) genes and is typically associated with colorectal, endometrial, and ovarian cancer. In this context, we describe the investigation and management of a 45-year-old female patient with a known family history of ovarian cancer and myocardial infarction who developed clinical features of hypercortisolism.

Endocrine evaluation demonstrated elevated cortisol and ACTH levels. The MRI revealed a microadenoma for which the patient underwent repeat TSS and Gamma Knife radiosurgery, leading to resolution of the pituitary adenoma. Subsequently, endometrioid adenocarcinoma was diagnosed, and in view of the family history, an underlying genetic disorder was suspected and later confirmed as LS with a pathogenic MSH6 variant, representing only the second reported case of MSH6-associated pituitary adenoma. Surgical management included hysterectomy with bilateral salpingo-oophorectomy, aortopelvic lymphadenectomy, and omental biopsy. This case underscores the importance of early diagnosis and lifelong monitoring in CD to reduce mortality from uncontrolled hypercortisolism, while highlighting a possible association between LS and ACTH-secreting pituitary adenomas, particularly those with MSH6 mutations.

## Introduction

Cushing’s disease (CD) arises from an adrenocorticotropic hormone (ACTH)-secreting pituitary adenoma that drives cortisol overproduction by the adrenal glands. It is associated with an increased risk of death, largely attributed to cardiovascular and infectious complications, and affects 1.8 to 2.6 individuals per million population annually [1]. Symptoms such as obesity, hypertension, and menstrual irregularities are prevalent in the average population and may complicate timely diagnosis [2]. Transsphenoidal surgery (TSS) is the first-line treatment for CD; however, 5% to 35% of patients treated with TSS experience recurrence of the disease. In cases of postoperative persistence or recurrence, treatment modalities include repeat TSS and pituitary radiotherapy [3].

Lynch syndrome (LS) is an autosomal dominant (AD) disorder caused by mutations of the mismatch repair (MMR) genes, which encode proteins critical for DNA repair. While LS is most commonly associated with colorectal, endometrial, and ovarian cancers, several case reports and a nationwide study conducted in Sweden have suggested a higher prevalence of pituitary adenomas in patients with LS [4,5]. While CD and LS appear to be distinct conditions, emerging evidence suggests a potential association.

We report a case of recurrent ACTH-secreting pituitary microadenoma in a patient with MSH6-associated LS. To the best of our knowledge, it is only the second reported case of MSH6-related pituitary adenoma, adding to the growing evidence of an association between these two conditions.

## Case presentation

In July 2008, a 45-year-old Czech woman presented to the internal medicine department with a nine-month history of facial redness, abdominal bloating, and decreased cold tolerance. Her medical history included type 2 diabetes mellitus treated with metformin, hypertension treated with amiloride/hydrochlorothiazide, combined hyperlipoproteinemia, and euthyroid autoimmune thyroiditis monitored annually. Family history was notable for a father who died of myocardial infarction at age 48, a mother who died of ovarian cancer at age 54, and a paternal aunt who died of a malignancy, most likely of gynecologic origin. The patient had two children, a 21-year-old son and a 14-year-old daughter, both delivered by cesarean section. The patient's menstrual history was notable for menarche at age 15 and regular cycles until two months before presentation, when irregularities developed.

On physical examination, cushingoid facies, plethora, central obesity, and suspected extremity muscle atrophy were noted. The thyroid was mildly enlarged. Blood pressure was 170/100 mmHg, heart rate 80 beats/min, and body mass index 31 kg/m². Initial laboratory evaluation showed hyperglycemia with glucose at 8 mmol/L (144 mg/dL) and prolactin within the female reference range. Hypercortisolism was confirmed by markedly elevated morning cortisol of 1067 and 1435 nmol/L (38.7 and 52.0 µg/dL) (reference range ≤624 nmol/L; ≤22.6 µg/dL) and increased 24-hour urinary free cortisol of 1148 nmol/24 h (416 µg/24 h) (reference range ≤1112 nmol/24 h; ≤403 µg/24 h). Thyroid ultrasound showed a mild multinodular goiter consistent with chronic thyroiditis with more prominent nodularity in the left lobe. Adrenal CT was unremarkable.

**Figure 1:**
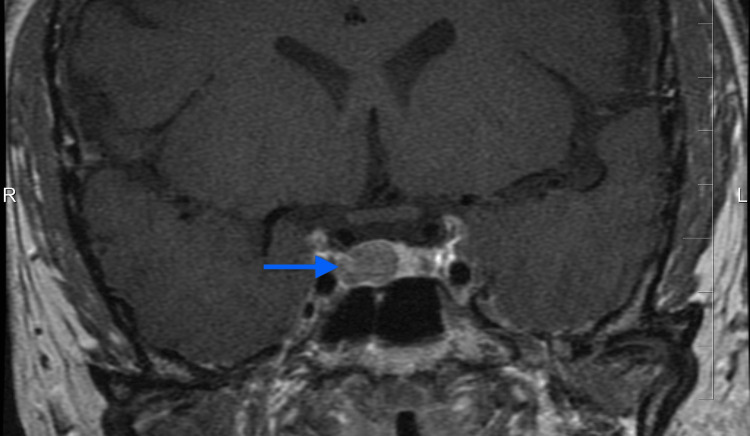
Preoperative pituitary MRI demonstrating a right-sided microadenoma (blue arrow)

Given the elevated cortisol levels, unremarkable CT findings, and physical evaluation concerning for CD, the patient was referred to a tertiary endocrinology center. Additional biochemical testing was performed and is summarized in Table 1. Electrocardiography showed sinus rhythm at 96 bpm, PR interval 160 ms, QRS duration 80 ms, and QTc 460 ms within normal limits for her sex and age. Antihypertensive, lipid-lowering, and steroidogenesis-inhibiting therapies were initiated with amlodipine 5 mg once daily, telmisartan 80 mg once daily, atorvastatin 10 mg daily, and ketoconazole 200 mg four times daily. A restrictive diet low in saturated fat and simple sugars was also recommended. Postcontrast T1-weighted MRI revealed a right-sided 8 mm x 6 mm hypoenhancing mass with leftward deviation of the pituitary stalk, consistent with a microadenoma (Figure 1). The lesion corresponded to Hardy II-A [6] and Knosp grade I [7].

**Table 1:** Summary of laboratory and endocrinological findings ACTH: Adrenocorticotropic hormone, HbA1C: Glycated hemoglobin

Diagnostic test	Parameter	Value (SI)	Value (US)	Reference range
Biochemistry	Potassium	4.2 mmol/L		3.5-5.1 mmol/L
	Glucose	6.80 mmol/L	122.52 mg/dL	3.9-5.5 mmol/L 70-99 mg/dL
	HbA1c	6.6%		<5.7 %
Endocrinology	ACTH	99.3 ng/L	99.3 pg/mL	10-60 ng/L 10-60 pg/mL
	ACTH (repeat)	66.7 ng/L	66.7 pg/mL	
	Serum cortisol 07:00	914 nmol/L	33.1 µg/dL	180-650 nmol/L 6.5-23.6 µg/dL
	Serum cortisol 13:00	704.8 nmol/L	25.5 µg/dL	
	Serum cortisol 19:00	791.2 nmol/L	28.7 µg/dL	
	Serum cortisol 01:00	635.8 nmol/L	23.0 µg/dL	
	Serum cortisol 07:00 (next day)	795 nmol/L	28.8 µg/dL	

**Figure 2:**
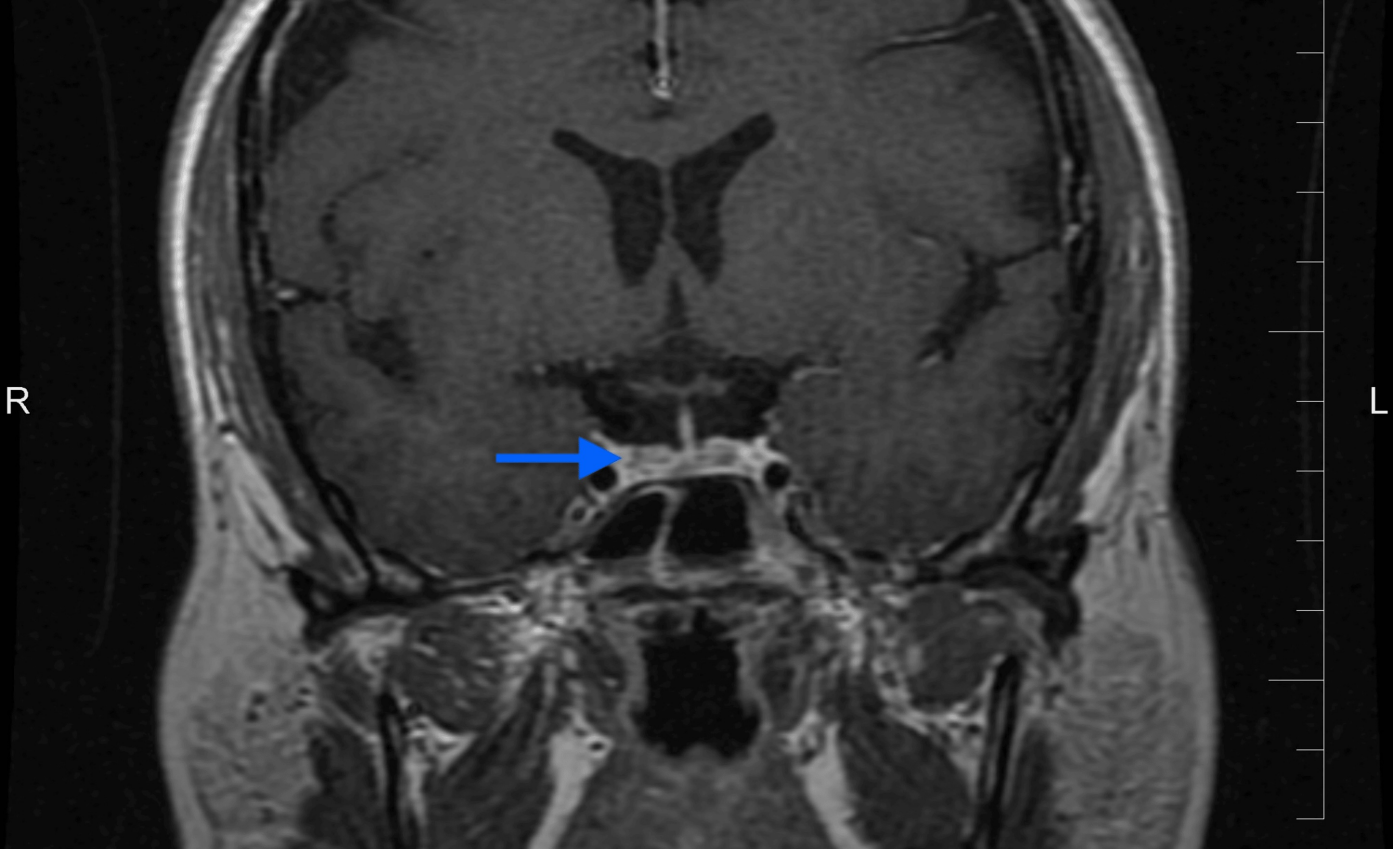
Coronal T1-weighted post-contrast MRI demonstrating postoperative changes at the prior adenoma resection site (blue arrow)

Due to the elevated ACTH levels and imaging findings diagnostic of a pituitary adenoma, the patient was referred to the neurosurgery department and underwent TSS. A gross total resection was achieved (Figure 2), and her postoperative course was uncomplicated. Postoperatively, the patient continued her standard medications for comorbid conditions. At three months after surgery, she was asymptomatic, and an MRI demonstrated no evidence of residual adenoma. Annual follow-up was arranged, with education provided to monitor for symptoms of recurrence.

**Figure 3:**
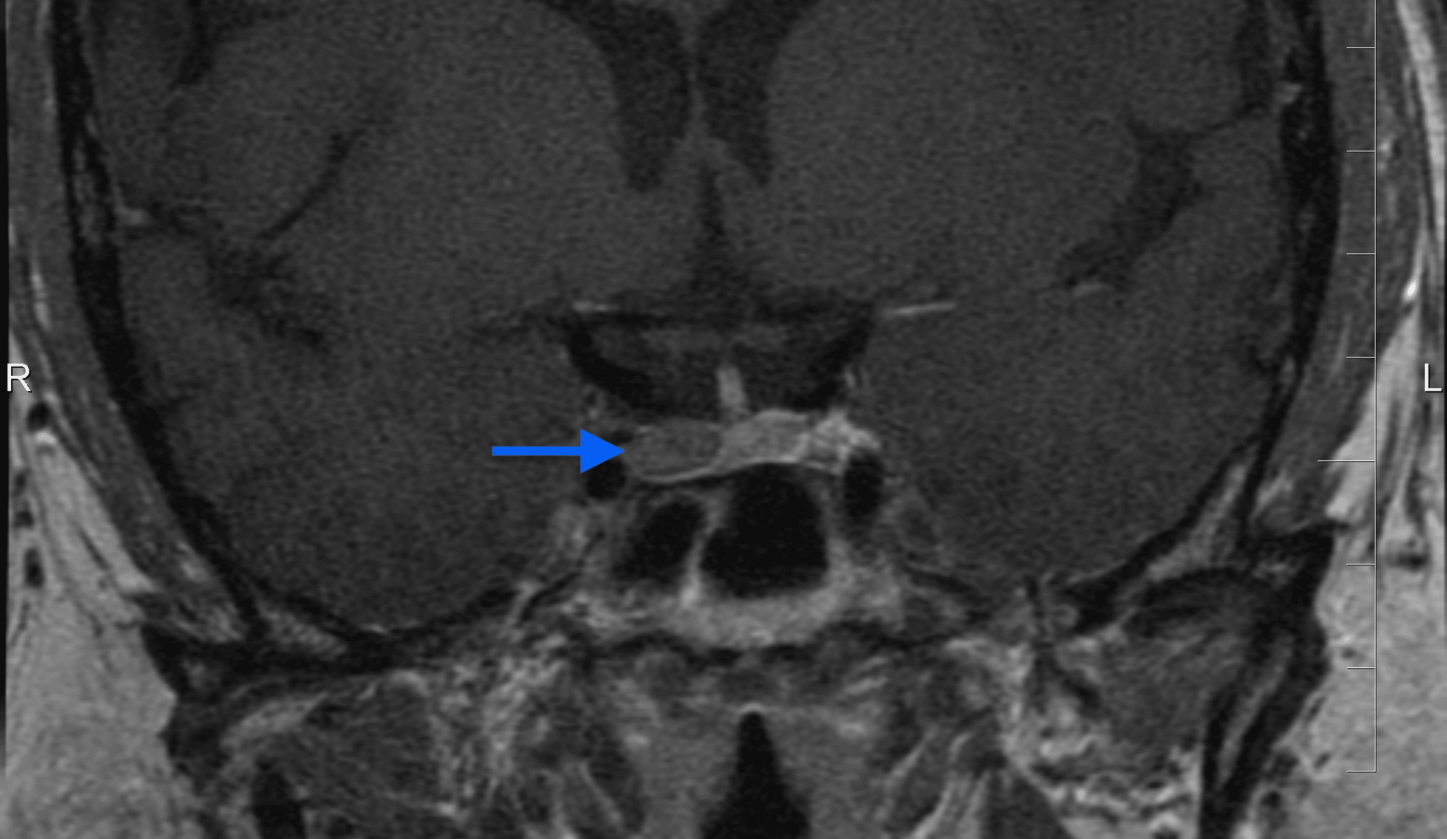
MRI evidence of pituitary adenoma recurrence (blue arrow)

Six years later, in 2014, a recurrent pituitary adenoma causing CD was confirmed by MRI (Figure 3), and the patient underwent repeat TSS. Early postoperative cortisol on day six was elevated at 680 nmol/L; however, reoperation was not pursued. Subsequent testing demonstrated cortisol values below the reference range, consistent with expected suppression following TSS, while other pituitary hormone values remained within normal limits. She was discharged on hydrocortisone 20 mg daily, and follow-up was arranged.

**Figure 4:**
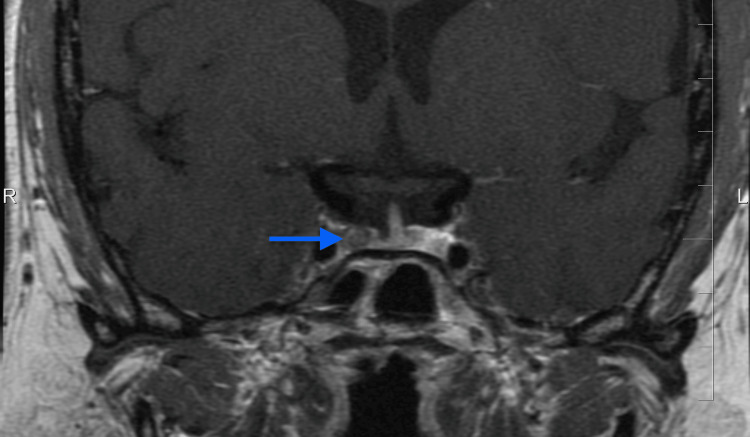
MRI demonstrating a second recurrence of pituitary adenoma (blue arrow)

In January 2018, an MRI revealed an 8 x 9 x 7 mm right-sided recurrent pituitary microadenoma (Figure 4). The patient underwent gamma knife radiosurgery, receiving a prescribed dose of 35 Gy to the 50% isodose line. Postoperatively, ketoconazole 200 mg twice daily was initiated but discontinued after eight days due to intolerance, including weakness, arthralgia, cold intolerance, diarrhea, and facial skin desquamation. At a follow-up in May 2018, no clinical or biochemical evidence of hypercortisolism was found. Morning cortisol was 214 nmol/L (≈ 7.8 µg/dL). Two 24-hour urinary free cortisol collections measured 23.4 nmol/day and 25.8 nmol/day (≈ 8.5 and 9.4 µg/day,) below the laboratory reference of 38 to 208 nmol/day (≈ 13.8-75.4 µg/day), consistent with postoperative suppression.

**Figure 5:**
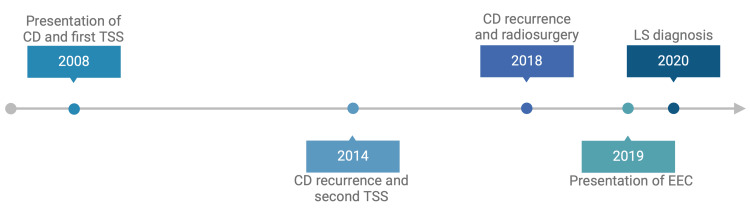
Timeline of events CD: Cushing's disease, TSS: transsphenoidal surgery, EEC: Endometrioid endometrial carcinoma, LS: Lynch syndrome Timeline created using BioRender (BioRender, Toronto, ON, CAN)

Endometrioid endometrial carcinoma was diagnosed in November 2019 (Figure 5). The patient subsequently underwent total hysterectomy with bilateral salpingo-oophorectomy, pelvic and para-aortic lymphadenectomy, and omental biopsy. Final pathology showed no nodal or omental metastases. The tumor demonstrated microsatellite instability (MSI), prompting germline testing for LS. Adjuvant vaginal cuff brachytherapy was delivered in six 4.0 Gy fractions, prescribed to a 0.5 cm depth. Germline analysis confirmed LS due to a heterozygous deletion in the MSH6 gene (c.3053delT), resulting in a frameshift and premature stop codon (p.Leu1018Profs*2). The patient’s current recommendations and periodic surveillance are summarized in Table 2.

**Table 2:** Current recommendations and periodic surveillance schedule CD: Cushing’s disease

Investigation/Examination	Frequency	Notes
Whole-body physical examination by a physician	Once per year	Includes skin check, basic neurological exam, and thyroid assessment (due to additional tumor risk)
MRI of the pituitary region	Every two years or earlier if symptoms arise	Monitoring for CD
Colonoscopy	Every two years, or annually if pathology is detected	Fecal occult blood test in between
Gastroscopy	Every three to four years or earlier if symptoms arise	
Small bowel evaluation	In case of unexplained GI symptoms not clarified by colonoscopy/gastroscopy	
Abdominal ultrasound	Once per year	Routine abdominal cancer screening
Urinalysis (chemical + sediment) and urological examination	Once per year	Malignancy screening for the urinary tract
Tumor marker testing	As indicated by the oncologist	
Mammography	Once per year	Additionally, a monthly breast self-examination
Lifestyle recommendations	Ongoing	Diet rich in fruit/vegetables, no smoking, regular exercise, stress reduction, limit toxins/sun

## Discussion

The patient developed cushingoid features in 2008. Biochemistry confirmed elevated ACTH, and MRI demonstrated a pituitary microadenoma consistent with ACTH secretion, establishing a diagnosis of CD. She underwent TSS in 2008 but experienced two recurrences, first in 2014 and again in 2018, the latter treated with stereotactic radiosurgery. In 2019, she was diagnosed with endometrioid adenocarcinoma of the endometrium. Germline testing identified a pathogenic MSH6 variant, confirming LS. Given the recognized risk of relapse after TSS, she entered guideline-directed surveillance. To our knowledge, this is only the second reported case of an MSH6 germline-associated pituitary adenoma.

Recurrence after TSS for CD is reported in 5% to 35% of patients, warranting lifelong monitoring in accordance with current guidelines [3]. For long-term surveillance, late-night salivary cortisol (LNSC) is the most sensitive screen for recurrence, with urinary free cortisol (UFC) and low-dose dexamethasone suppression also serving as possible monitoring tests [8]. In this case, LNSC and ACTH were measured postoperatively, and long-term surveillance consisted of morning serum cortisol assessments and interval MRI. Following the second recurrence, stereotactic radiosurgery, an accepted second-line option for persistent/recurrent disease, was performed with favorable early control. The principal late toxicity of pituitary radiotherapy is hypopituitarism; nevertheless, no hypopituitarism has developed to date [1,9]. This clinical course underscores the importance of guideline-concordant, lifelong biochemical screening after TSS and the role of targeted radiotherapy when repeat surgery is not curative.

Endometrial cancer is the most common malignancy in LS, as in our patient. Protein product MSH6 of the MSH6 gene, along with MSH2, plays a vital role in forming a MutSα heterodimeric complex, which recognizes mismatch errors during DNA replication [10]. Our patient carried a heterozygous deletion in the MSH6 gene, causing a frameshift and premature stop codon, producing a truncated, nonfunctional protein. Such a pathogenic germline variant establishes the diagnosis of LS. Subsequent mismatch repair deficiency arises with a somatic mutation of the second allele during the patient’s lifetime, the so-called 'second-hit' of the two-hit hypothesis [11].

Defective MMR allows DNA replication errors to accumulate, producing MSI with frequent frameshifts. This process in coding genes potentially leads to a loss of function in key regulator genes, conferring a clonal growth advantage [12,13]. Clinically, MSI therefore prompts evaluation for LS, most notably in colorectal and endometrial cancers. By analogy, the same mutational mechanism could plausibly contribute to tumorigenesis in this patient’s pituitary adenoma, although direct evidence remains limited.

Pituitary neoplasms have also been described in patients with LS, and the studies suggesting such affiliation are listed in Table 3. In the published cases, most tumors arise in the setting of MLH1 and MSH2 gene mutations and tend to be macroadenomas or carcinomas. Several show loss of MMR protein expression within the tumor, supporting the LS-related mechanism of pathogenesis. It has been proposed that diminished MSH2/MSH6 impairs ATR-Chk1-mediated checkpoint control, fostering pituitary adenoma growth through faster cycling and reduced apoptosis [14]. Our patient carries a germline MSH6 variant; however, MMR protein expression or MSI assessment in the pituitary microadenoma itself was not performed, limiting conclusions regarding MMR-driven tumorigenesis in this lesion. 

**Table 3:** Published human case reports linking LS to pituitary tumors ACTH: Adrenocorticotropic hormone, LS: Lynch syndrome, MSI: Microsatellite instability; MMR: Mismatch repair, TSS: Transsphenoidal surgery, CD: Cushing’s disease

Author and year of publication	LS germline mutation	Tumor sequencing	Pituitary tumor type	Age and sex	Notes
Uraki et al. (2017) [15]	MLH1 (germline); MSH6 (somatic in tumor)	MLH1 and MSH6 loss	ACTH-secreting pituitary adenoma (CD) – atypical, invasive	68F	MLH1-mutated LS; developed aggressive corticotroph adenoma. Tumor sequencing revealed loss of MLH1 and MSH6.
Bengtsson et al. (2017) [4]	LS (not specified, registry-based)	MSH2 and MSH6 loss	Corticotroph pituitary carcinoma (ACTH-secreting)	51M	Case report of an LS patient with ACTH-producing pituitary carcinoma, plus nationwide cohort (910 LS patients) showing three pituitary tumors vs ~1 expected. Highlights the possible association of LS and pituitary tumors.
Voisin et al. (2019) [16]	MSH2 + MSH6	MSH2 and MSH6 loss	Undifferentiated carcinoma of the sella (nonfunctioning)	56F	Dual MMR mutations (germline MSH2/MSH6), aggressive undifferentiated sellar carcinoma, metastases to lung and bone.
Loughrey et al. (2021) [17]	MSH2	MSH2 loss	ACTH-secreting pituitary adenoma – invasive macroadenoma	42M	ACTH-producing macroadenoma secondary to a germline MSH2 mutation as first LS manifestation. Persistent hypercortisolism; treated with radiosurgery.
Teuber et al. (2024) [18]	MSH2	Not mentioned	Prolactin-secreting pituitary adenoma – aggressive lactotroph	56F	Patient with germline MSH2 mutation LS developed an invasive prolactinoma. Concludes that aggressive, treatment-refractory tumor recurrence should prompt germline MMR testing; if positive, broaden cancer screening and adjust management.
Present case (2025)	MSH6 (germline) c.3053delT → p.Leu1018Profs*2	Has not been performed	ACTH-secreting pituitary microadenoma (CD); recurrent	45F	CD presentation in 2008; TSS 2008 and 2014 with persistence/recurrence; gamma knife radiosurgery (2018) with biochemical control; endometrioid adenocarcinoma (2019) MSI-high; germline MSH6 confirmed (2020); ongoing surveillance.

Importantly, in a Swedish nationwide cohort of 910 individuals with LS, three pituitary tumors were observed, whereas only one would have been expected, indicating an excess risk. Two cases presented with a pituitary tumor as the initial manifestation, underscoring that pituitary disease can be the initial presenting feature in LS. Moreover, in one patient, tumor-level MMR and MSI testing were available. The tumor showed loss of MSH2 and MSH6 with retention of MLH1 and PMS2, consistent with the patient’s germline MSH2 mutation, and exhibited an MSI-low phenotype [4].

Limitations of this case report in terms of the causal relationship of the MMR gene mutation and pituitary adenoma result from incomplete genetic profiling of the second-hit mutation in the pituitary adenoma due to no suspicion of a genetic disorder at the time. Therefore, the evidence is purely suggestive, and more data are necessary to confirm or refute this hypothesis. Nevertheless, this case raises awareness of pituitary adenoma occurrence in patients with LS and draws attention to the need for larger cohort studies to clarify the association.

## Conclusions

This case illustrates recurrent pituitary adenomas in a woman with LS due to the MSH6 gene mutation. It reinforces the need for lifelong monitoring of recurrence, which is in line with current guidelines. In patients with LS who develop atypical, aggressive, or recurrent pituitary tumors, assessment of tumor MMR status using immunohistochemistry and MSI analysis may help clarify pathogenesis and guide next steps in care. Currently, routine genetic testing is not recommended for sporadic pituitary adenomas; however, larger studies are needed to confirm or refute a causal link between LS and pituitary adenomas.
